# Longitudinal study of microphthalmia in connexin 50 knockout mice using spectral-domain optical coherence tomography

**DOI:** 10.3389/fopht.2024.1387961

**Published:** 2024-05-03

**Authors:** Taishi Painter, Chenxi Ou, Xiaohua Gong, Chun-hong Xia

**Affiliations:** Herbert Wertheim School of Optometry and Vision Science Program, University of California, Berkeley, Berkeley, CA, United States

**Keywords:** optical coherence tomography, lens growth, cataract, connexin 50, knockout

## Abstract

Connexin 50 (Cx50) mediated signaling is essential for controlling the lens growth and size. Cx50 mutations cause microphthalmia, smaller lenses, and cataracts in humans and animals. These ocular defects have never been investigated in live Cx50 mutant mice by using non-invasive imaging techniques. Here, we report a longitudinal study of the ocular defects in Cx50 knockout (Cx50KO) mice from the ages of 3 weeks to 12 months by using spectral-domain optical coherence tomography (SD-OCT). The anterior chamber depth (ACD), lens thickness (LT), vitreous chamber depth (VCD), and axial length (AL) were measured along the visual axis and adjusted with corresponding refractive indices. The SD-OCT image data confirm age-related reductions of LT and AL in live Cx50KO mice compared to age-matched wild-type (WT) controls, and the reduction values are comparable to the *in vitro* measurements of Cx50KO eyeballs and lenses reported previously. Moreover, reductions of ACD were observed in Cx50KO mice at all ages studied while VCD changes are statistically insignificant in comparison to the WT controls. Therefore, Cx50KO’s microphthalmia with small lens is selectively associated with delayed ACD development but not the vitreous formation. This work supports the notion that lens size and/or growth is important for anterior chamber development.

## Introduction

The ocular lens growth, transparency, and homeostasis rely on distinct functions of gap junction channels formed by connexins including connexin 46 (Cx46) or Gja3 and Cx50 or Gja8 (1–6). Cx50 knockout mice display smaller lenses with mild nuclear cataracts (7, 8), indicating connexin 50 is essential for lens growth control. Previous studies on mouse lens growth have relied on the *in vitro* measurements of the size and weight of enucleated eyeballs and lenses from euthanized mice (6–14). The advance of non-invasive spectral-domain optical coherence tomography (SD-OCT) allows for the acquisition of a single image that reaches a maximum imaging depth sufficient to capture the entire axial length of the mouse eye, thus providing a valuable tool for longitudinally monitoring the eye/lens growth and cataract formation in live mice.

Optical coherence tomography is one of the standard tools to image and measure the retinal thickness (15) and the anterior chamber for angle closure (16) for the diagnosis and management of some eye diseases in ophthalmologic clinics. OCT has also been used in animal models to measure biometric properties of the eye longitudinally (17–19), it provides an effective tool to determine anterior segment features, the lens, and the retina in different mouse eye models *in vivo* (20–23). Here, we have used OCT to carry out a longitudinal study of the eye and lens growth in live Cx50KO mice.

Cx50 is restrictively utilized in the lens but not in the other parts of the eye (7, 24, 25). Cx50KO and mutant mice develop small lenses containing nuclear cataracts and microphthalmia (7–9, 25–28). The loss of Cx50 function suppresses the proliferation and differentiation of lens epithelial cells to lead to a smaller lens. Small sized lenses seem to directly cause microphthalmia in Cx50KO mice. Human Cx50 mutations cause cataracts, microphthalmia, microcornea, and anomalies of iris in patients (29–37). Therefore, Cx50KO mice provide a valuable model for investigating different ocular structures such as measuring anterior chamber depth (ACD), lens thickness (LT), vitreous chamber depth (VCD), and visual axial length (AL) along the optical axis in the eyes of live mice by SD-OCT. The precise measurement of anatomic structures in the eye can be determined in the optical axis and adjusted with corresponding refractive indices (20). We have characterized the longitudinal changes of ACD, LT, VCD, and AL in both wildtype (WT) and Cx50KO mice from the ages of three weeks to 12 months.

## Materials and methods

### Animals

Wild-type (WT) mice and Cx50 knockout (Gja8^tm1^) mice (7) at the C57BL/6J background were used for the study. Mice were housed under a 12-hour light cycle with normal food and water. All experimental procedures were approved by the Animal Care and Use Committee (ACUC) at the University of California, Berkeley (Berkeley, CA, USA), and were conducted in accordance with the ARVO Statement for the Use of Animals in Ophthalmic and Vision Research.

### Optical coherence tomography

The Leica Envisu R4310 spectral domain OCT system (Bioptigen, Leica Microsystems Inc.) with a center wavelength of 840nm and a telecentric 10mm lens was used to image the eyes of live mice *in vivo*. Mice were anesthetized during the OCT imaging with an intraperitoneal injection of ketamine (80mg/kg) and xylazine (12mg/kg) diluted in 1x PBS (Phosphate buffer saline); mouse corneas were continuously hydrated using lubricating eye drops during and after imaging until the mice would wake up to prevent drying and maintain transparency. Three types of scans were acquired: rectangular 600 A-scans x 600 B-scans at 3.5mm by 3.5mm, rectangular 600 A-scans x 600 B-scans at 3mm by 3mm, and a radial 600 A-scans x 600 B-scans at a radius of 3mm. For each mouse eye, all three scans were taken for measurements. It took approximately 15 minutes to image both eyes of each mouse; the mouse was placed on a heated mat to stay warm until awake afterward.

**Figure 1:**
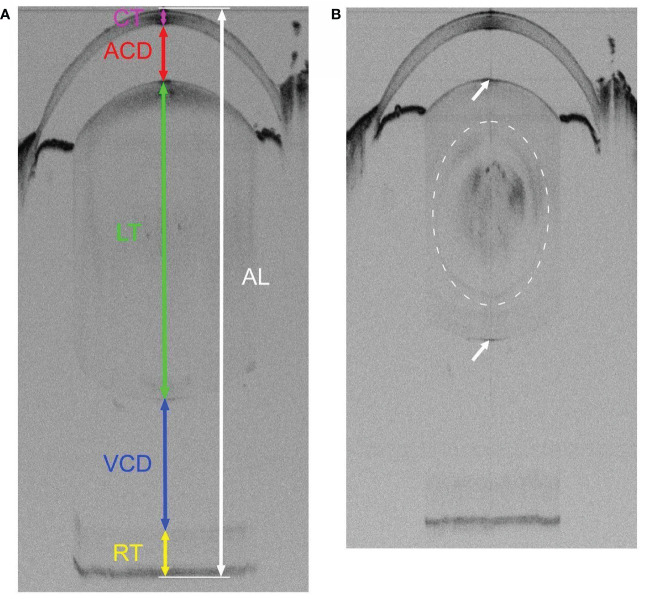
Representative SD-OCT whole eye images of 3-week-old WT **(A)** and Cx50KO **(B)** mice. OCT scan captures the entire visual axial length from the surface of the anterior cornea to the back of the posterior retina of the mouse eyeball. **(A)** The colored lines represent the measurements of different parts of ocular structures along the visual axis using the Bioptigen Envisu Software: corneal thickness (CT, magenta), anterior chamber depth (ACD, red), lens thickness (LT, green), vitreous chamber depth (VCD, blue), retinal thickness (RT, yellow), and visual axial length (AL, white). **(B)** A mild nuclear cataract, indicated by a dashed cycle, can be seen as noticeable diffused black areas in the Cx50KO lens core. Both anterior and posterior surfaces of the lens are indicated by arrows.

**Figure 2:**
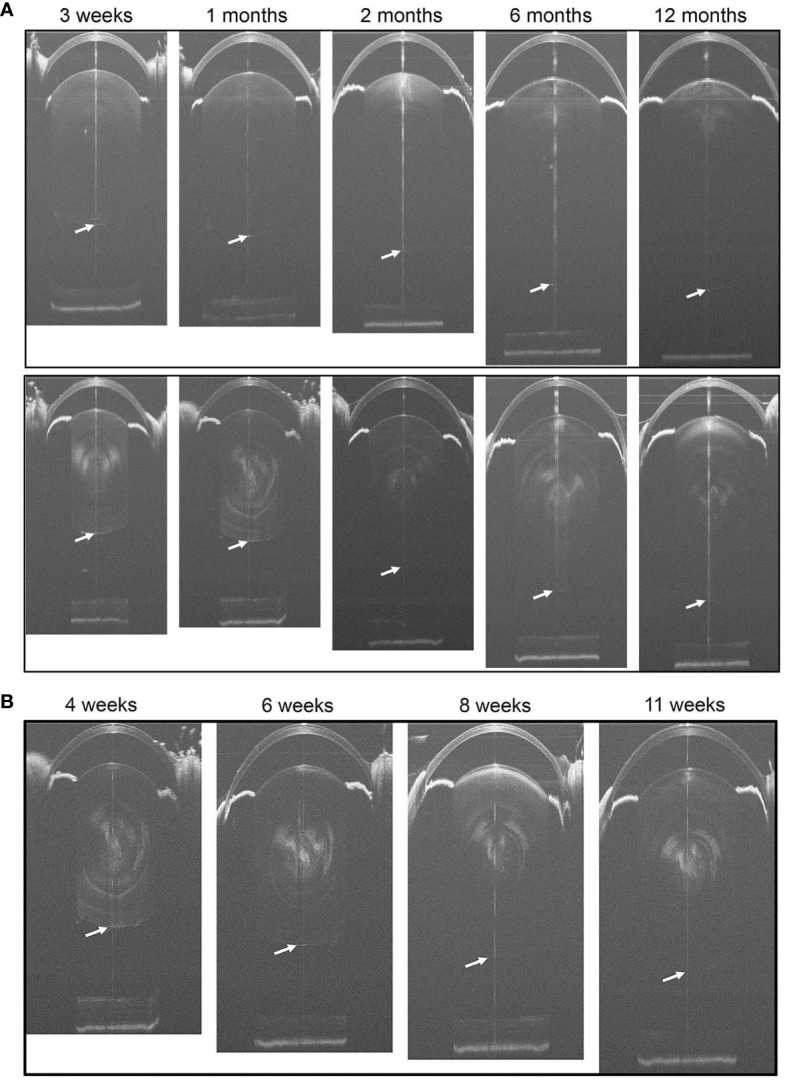
**(A)** Representative SD-OCT whole-eye images of WT (the upper panels) and Cx50KO (the lower panels) mice at different ages from 3 weeks to 12 months. Cataracts in the lens core of Cx50KO mice are clearly visible as opaque white areas. The lens posterior poles are indicated by the arrows. **(B)** Longitudinal SD-OCT eye images of the same eye of a KO mouse from 4 weeks to 11 weeks old. The posterior surfaces of lenses are indicated by white arrows.

Published images were processed using ImageJ/FIJI (NIH, Bethesda, MD, USA). Images were averaged over 10 frames to reduce noise. All OCT images shown (**Figures 1**, **2**) are from 3x3mm rectangular scans.

### Biometry and image processing

Measurements, mainly from the radial scans, were manually taken with calipers along the Purkinje line near the center of the pupil for each eye using the InVivoVue 2.4 OCT Management Software. The following measurements were obtained: corneal thickness (CT), anterior chamber depth (ACD), lens thickness (LT), vitreous chamber depth (VCD), retinal thickness (RT), and axial length (AL). Measurements were converted into geometric distances using corresponding refractive indices based on previous publications (11, 20): 1.4015 for the cornea, 1.3336 for the anterior chamber, 1.45 for the lens thickness, 1.3329 for the vitreous chamber, and 1.38 for the retina. The geometric distance for axial length was calculated by summing CT, AC, LT, VC, and RT.

### Statistical analysis

The Two-sided Wilcoxon Rank Sum test was performed to determine if there is a significant difference between the two different genotypes across all age groups, while a One-sided Wilcoxon Rank Sum test was performed to compare the change of biometric measurements for one genotype over time.

## Results

The non-invasive SD-OCT imaging system can capture the entire axial length of a mouse eye. The SD-OCT image of a 3-week-old WT eye shows the cornea, the anterior chamber, the lens, the vitreous chamber, and the retina (**Figure 1A**); and the CT, ACD, LT, VCD, and RT are indicated with colored lines in **Figure 1A**. The SD-OCT image of a 3-week-old Cx50KO eye also displays dark areas in the lens core (**Figure 1B**), which correspond to the mild nuclear cataract (7, 8).

**Figure 3:**
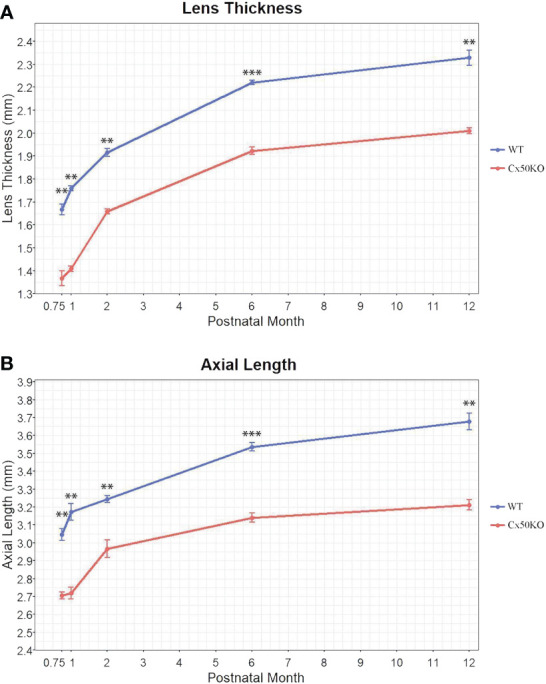
Comparisons of lens thickness **(A)** and visual axial length **(B)** of WT and Cx50KO mice at the ages of 3 weeks, 1 month, 2 months, 6 months, and 12 months old. Data points represent the mean ± SD (n = 4-12 per data point). Two-sided Wilcoxon test was performed for statistical analysis, ** *P* < 0.01, *** *P* < 0.001.

To compare the lens and eye growth between the WT and Cx50KO mice during postnatal development, SD-OCT images were acquired from mice at the ages of 3 weeks, 1 month, 2 months, 6 months, and 12 months (**Figure 2**). **Table 1** presents all measurements (mean ± SD, n = number of imagined eyes) for lens thickness (LT), axial length (AL), anterior chamber depth (ACD), and vitreous chamber depth (VCD). The growth rates of the lens and the eye are reflected by the increases in the lens thickness and the axis length in the SD-OCT images, respectively, as mice aging (**Figure 3A**). In the WT mice, the steep increase of lens thickness is observed between 3 weeks to 1 month, the LT continues to increase until reaching the age of 2 months, from which the LT growth rate tapers off. The Cx50KO mice exhibit a similar growth rate compared to the WT, except for the period between 3 weeks and 1 month, when the growth curve is much flatter than the WT, suggesting a much slower growth of Cx50KO lens during this period. The AL growth curves (**Figure 3B**) also indicate a slower growth rate in Cx50KO between 3 weeks to 1 month. Overall, the Cx50KO mice show significant reductions of both LT and AL at all ages examined compared to the WT controls (**Figure 3**; **Table 2**). Moreover, the AL growth rate in Cx50KO mice obviously lags that of WT mice between 3 weeks to 1 month old (**Figure 3B**; **Table 2**). **Table 2** lists percentage change values for all measurements between Cx50KO and WT. In summary, the data reveal an obvious reduction in the sizes of the lens and the eye in Cx50KO mice at all ages.

**Table 1:** Measured values for lens thickness (LT), axial length (AL), anterior chamber depth (ACD), and vitreous chamber depth (VCD).

WT	Age	LT (mm)	AL (mm)	ACD (mm)	VCD (mm)
	3 weeks	1.667±0.024 (n=5)	3.046±0.033 (n=5)	0.311±0.020 (n=6)	0.729±0.018 (n=5)
	1 month	1.76±0.010 (n=4)	3.173±0.047 (n=4)	0.337±0.016 (n=5)	0.729±0.028 (n=5)
	2 months	1.916±0.017 (n=8)	3.245±0.020 (n=8)	0.361±0.010 (n=8)	0.650±0.025 (n=8)
	6 months	2.221±0.010 (n=8)	3.536±0.023 (n=8)	0.425±0.013 (n=8)	0.563±0.026 (n=8)
	12 months	2.328±0.033 (n=5)	3.678±0.047 (n=5)	0.452±0.021 (n=5)	0.533±0.036 (n=5)
Cx50KO	Age	LT (mm)	AL (mm)	ACD (mm)	VCD (mm)
	3 weeks	1.366±0.032 (n=10)	2.706±0.019 (n=6)	0.265±0.018 (n=13)	0.743±0.040 (n=6)
	1 month	1.409±0.011 (n=12)	2.720±0.033 (n=12)	0.283±0.014 (n=12)	0.688±0.022 (n=12)
	2 months	1.659±0.011 (n=6)	2.966±0.0493 (n=4)	0.325±0.011 (n=6)	0.609±0.030 (n=4)
	6 months	1.923±0.015 (n=8)	3.140±0.026 (n=8)	0.342±0.010 (n=8)	0.547±0.019 (n=8)
	12 months	2.010±0.012 (n=8)	3.212±0.029 (n=8)	0.351±0.014 (n=8)	0.507±0.013 (n=8)

The data are shown as mean ± standard deviation (n = number of individual eyes measured).

**Table 2:** Percentage change of LT, AL, ACD, and VCD in Cx50KO mice compared to WT controls.

Age	Δ LT (%)	Δ AL (%)	Δ ACD (%)	Δ VCD (%)
3 weeks	-18.05±2.27% **	-11.14±1.15% **	-14.85±7.99% ***	1.88±6.07%
1 month	-19.93±0.79% **	-14.29±1.64% **	-16.13±5.77% **	-5.56±4.72% **
2 months	-13.41±0.96% **	-8.58±1.62% **	-9.85±3.85% **	-6.28±5.90%
6 months	-13.40±0.78% ***	-11.21±0.95% ***	-19.63±3.49% ***	-2.94±5.60%
12 months	-13.68±1.33% **	-12.68±1.35% **	-22.35±4.84% **	-4.72±6.94%

Negative values are percentage reduction, positive values are percentage increase. The data are shown as mean ± standard deviation. Two-sided Wilcoxon test was performed for statistical analysis, *P < 0.05, **P < 0.01, ***P < 0.001.

**Figure 4:**
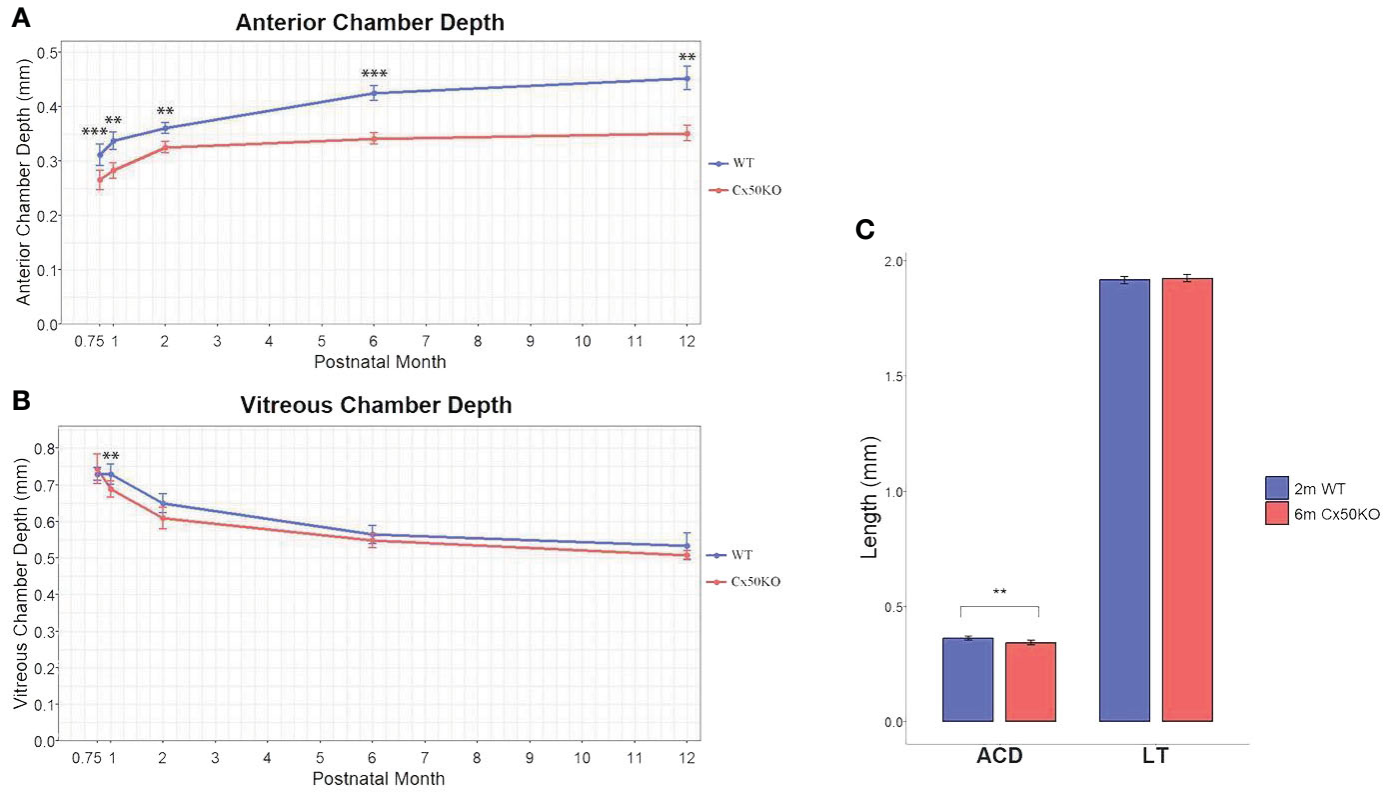
**(A)** Comparison of anterior chamber depth in WT and Cx50KO mice from 3 weeks to 12 months old. Data points represent mean ± SD (n = 5-13 per data point)). Two-sided Wilcoxon test was performed for statistical analysis, **P < 0.01, ***P < 0.001. **(B)** Comparison of vitreous chamber depth in the WT and Cx50KO mice from 3 weeks to 12 months old. Data points represent mean ± SD (n = 4-12 per data point). Two-sided Wilcoxon test was performed for statistical analysis. There are no significant VCD changes between WT and Cx50KO mice, except at 1 month old, **P < 0.01. **(C)** Bar graphs showing the comparisons of anterior chamber depth (ACD) and lens thickness (LT) between 6-month-old Cx50KO and 2-month-old WT mice. Data are mean ± SD (n = 8), Two-sided Wilcoxon test was performed for statistical analysis, ** *P* < 0.01 for ACD. No significant difference for LT.

The SD-OCT data show significantly reduced anterior chamber depth in Cx50KO eyes compared to WT controls at all ages (*P* < 0.01, Two-sided Wilcoxon test, n = 5-13 eyes per data point) (**Figure 4A**). Moreover, the ACD differences between Cx50KO and WT increase from ~15% at 3 weeks old to ~22% at 12 months old. In the Cx50KO eyes, the ACD values reach a plateau after the age of 2 months, as the values remain almost the same from 2 months until 12 months. Thus, SD-OCT image data indicate a specific suppression of ACD expansion in Cx50KO mice after 2 months. In both Cx50KO and WT mice, the VCD reduces as the mice age (**Figure 4B**) and displays a reduction of about 27% from 1 month to 12 months (One-sided Wilcoxon test, *P* < 0.01, n = 5-8 eyes). There are no significant VCD differences between WT and Cx50KO mice at all ages examined (Two-sided Wilcoxon test, *P* > 0.05, n = 4-12 eyes per data point), except at 1 month when the Cx50KO mice display ~6% reduction of VCD (Two-sided Wilcoxon test, *P* < 0.01; **Table 2**). From 3 weeks to 1 month, a reduction of VCD occurs in Cx50KO mice while the value stays similar in WT mice (**Figure 4B**); this probably leads to the significant difference in VCD between Cx50KO and WT at 1 month old. To address whether the lens size determines the anterior chamber depth, we have selectively examined the eyes of 6-month-old Cx50KO mice and 2-month-old WT mice that have the same lens thickness (**Figure 4C**). The bar graphs show that the LT values are about the same between 6-month-old Cx50KO and 2-month-old WT (Two-sided Wilcoxon test, *P* = 0.49), but the ACD of Cx50KO mice is about 5% reduced than that of WT controls (Two-sided Wilcoxon test, *P* < 0.01).

## Discussion

The SD-OCT imaging data precisely reveal the longitudinal growth changes of anterior chamber depth, lens thickness, vitreous chamber depth, and visual axial length between WT and Cx50KO eyes from the ages of 3 weeks to 12 months *in vivo*. We applied an average 1.45 refractive index for mouse lenses at all ages according to the previous OCT study of mouse lenses (20), assuming the lens is a sphere and the gradient of refractive index (GRIN) of the lens is parabolic; we did not consider minor GRIN differences among wild-type lenses at different ages or cataract formation in Cx50KO lenses. A previous study reports that the lens max refractive indexes are 1.43 to 1.54 from 2-week-old to 2-year-old lenses, and the 6-week-old lens has the max refractive index of 1.5 with an average refractive index of about 1.45 (38). Thus, the application of the 1.45 average refractive index over an age-dependent GRIN causes minor differences, about 2-4%, in 3-week-old or 1-year-old lenses. Such minor differences are neglectable in this study comparing the significant differences between age-matched wild-type and Cx50KO lenses. The reduction of the lens thickness and axial length in Cx50KO eyes *in vivo* is consistent with previous studies of lens weight and eye size *in vitro* (6–8, 10). Similar growth trajectory of the lens and the eye between Cx50KO and WT mice at the ages of 3 weeks to 12 months further indicates that disruption of Cx50 mostly impacts early development before 3 weeks, which is supported by the fact that Cx50 deletion inhibits lens epithelial cell proliferation within the first postnatal week when the maximum mouse lens growth occurs (6, 8, 39). However, it is difficult to perform the OCT imaging in the small eyes of Cx50KO mice before 3 weeks and SD-OCT measurement is not applicable until the mouse opens its eyelid around postnatal day 14. Therefore, this work evaluates only the eye and lens growth after the age of 3 weeks *in vivo*.

For lens growth, our SD-OCT data show that the steepest increase of lens thickness occurs between 3 weeks to 1 month, and the LT growth rate of Cx50KO is lower than the WT control during this period. The LT growth gradually slows down after 1 month, and the LT growth rate is comparable between Cx50KO and WT after 2 months old. Based on the lens thickness curve, the Cx50KO eyes exhibit a similar growth rate compared to the WT, except for the period between 3 weeks and 1 month, when the growth curve is much flatter than the WT, suggesting a much slower growth of Cx50KO lens compared to the WT control during this period.

If we use the lens thickness as the spherical diameter to calculate the lens volume, the Cx50KO lenses are approximately 40% smaller than the WT lenses at the age of 3 weeks; this reduction value is very similar to our previous data using dissected lens measurement *in vitro* (9).

The axial length growth rate of Cx50KO is drastically lower than WT control from 3 weeks to 1 month; an obviously faster AL growth rate was observed in Cx50KO compared to the WT control from 1 month to 2 months; then the AL growth rate of Cx50KO becomes slower than the WT after the age of 2 months. Therefore, SD-OCT data detects a unique difference in the AL growth rate between WT and Cx50KO. AL growth burst occurs before the age of 1 month in WT while Cx50 AL growth burst occurs at the ages between 1-2 months. The cause for such a unique difference in AL growth is probably related to the growth difference of anterior chamber depth between WT and Cx50KO. The ACD curves (**Figure 4A**) indicate that the ACD growth of Cx50KO reaches almost a plateau at 2 months old while the ACD of WT gradually increases at all ages. The ACD growth of WT mice starts to decrease after the age of 1 month, then maintains at a similar level between the ages of 1 month to 6 months and reduces slightly between 6 months to 12 months. In contrast, the ACD growth of Cx50KO shows a steady increase between 3 weeks and 2 months, then the growth becomes drastically slower after 2 months to 12 months. The ACD differences between Cx50KO and WT increase after the ages of 2 months until 12 months. Cx50 is restrictively expressed in the lens and Cx50KO directly affects postnatal lens size/growth. Therefore, anterior chamber growth defect in Cx50KO is the secondary effect of Cx50KO postnatal lens defects. Altered ACD in Cx50KO mice indicates that the growth rate of postnatal lens size affects ACD development in the anterior segment.

Our SD-OCT data precisely reveal the growth defects of LT, AL, and ACD in Cx50KO eyes *in vivo* at different ages. More importantly, this *in vivo* study reveals that postnatal lens growth is one of the key factors to regulate the development of anterior chamber formation. The comparison between 2-month-old WT and 6-month-old Cx50KO mice further reveals that even when the Cx50KO lens eventually reaches the same LT as the WT lens, the ACD of Cx50KO lens is still significantly reduced than that of WT (**Figure 4C**). This further supports a conclusion that the postnatal growth rate of the lens size is critical for the development of the anterior chamber. This conclusion is also supported by studies in teleost that show the lens is necessary for the development of the overall eye in the early stages of growth (40) and can explain disrupted anterior segments in both mice and humans with mutated Cx50 (26, 29–32, 34, 37). The mechanism underlying the coordinated growth regulation among LT, AL, and ACD is not well studied. Cx50KO mice may provide an invaluable model for investigating the regulatory mechanism of anterior chamber development. Future investigation will be needed to address how the lens size can promote or inhibit the formation of the anterior segment.

The vitreous chamber depth decreases from three weeks to 12 months in both WT and Cx50KO mice. Although human studies have reported VCD increases as aging (41–43), VCD reduction as mice age has been reported in previous studies (20, 23). The age-related VCD differences between humans and mice likely rely on the fact that the human lens is about 3% of the eyeball size while the mouse lens accounts for one-third of the eyeball size. Age-related VCD reduction is likely due to the increase in lens size in mice. Statistical analysis indicates that the VCD differences between Cx50KO and WT mice are insignificant. Overall, our data suggest that the vitreous is well formed in mice before the age of three weeks, Cx50 deletion seems to cause little impact on the vitreous development.

In summary, SD-OCT is a powerful system to precisely measure the biometric properties of WT and Cx50KO eyes in live mice at postnatal ages. This *in vivo* imaging study reveals unique and intricate growth defects in ACD and AL in Cx50KO mice, that cannot be characterized by *in vitro* approaches. Cx50KO mice may be a valuable model for understanding how the growth of the postnatal lens size regulates the anterior chamber formation and for further investigating the regulatory mechanism underlying anterior segment development and visual axis length development during aging.

## Data availability statement

The raw data supporting the conclusions of this article will be made available by the authors, without undue reservation.

## Ethics statement

The animal study was approved by the Animal Care and Use Committee (ACUC) at University of California, Berkeley. The study was conducted in accordance with the local legislation and institutional requirements.

## Author contributions

TP: Data curation, Formal analysis, Investigation, Methodology, Software, Validation, Visualization, Writing – original draft, Writing – review & editing. CO: Data curation, Formal analysis, Investigation, Methodology, Software, Validation, Visualization, Writing – review & editing. XG: Conceptualization, Funding acquisition, Resources, Writing – review & editing, Writing – original draft. C-hX: Conceptualization, Funding acquisition, Investigation, Project administration, Supervision, Validation, Writing – original draft, Writing – review & editing.
